# Corticosteroid Injections for Pain Relief and Range of Motion in Adhesive Capsulitis

**DOI:** 10.7759/cureus.90535

**Published:** 2025-08-19

**Authors:** Muhammad Mannan, Syed Muneeb Ali Shah, Zawar Ahmad, Muhammad Tayyab, Faisal Karim, Muhammad Zeeshan Akram, Rehan Raza Shan

**Affiliations:** 1 Orthopedic Surgery, University Hospitals Birmingham NHS Foundation Trust, Birmingham, GBR; 2 Trauma and Orthopedics, University Hospitals Birmingham NHS Foundation Trust, Birmingham, GBR; 3 Trauma and Orthopedic, MTI Khyber Teaching Hospital Peshawar, Peshawar, PAK; 4 Trauma and Orthopedics, Kettering General Hospital, Kettering, GBR; 5 Trauma and Orthopedics, Hayatabad Medical Complex Peshawar, Peshawar, PAK; 6 Trauma and Orthopedics, Ghurki Trust Teaching Hospital, Lahore, PAK

**Keywords:** adhesive capsulitis, corticosteroids, frozen shoulder, intra-articular injection, shoulder pain, visual analogue scale

## Abstract

Introduction and objective: Adhesive capsulitis (frozen shoulder) is a chronic, painful shoulder condition characterized by progressive stiffness and restricted range of motion. Despite its prevalence, there is limited evidence, particularly in our regional population, regarding the optimal timing, dosage, and technique of corticosteroid injection to achieve maximal pain relief and functional recovery. The objective of this study was to evaluate the efficacy of intra-articular corticosteroid injections in patients with adhesive capsulitis, as measured by pain reduction and improvement in range of motion (ROM). Efficacy was defined as a reduction in pain exceeding the minimal clinically important difference (MCID) of 1.5 points on the visual analog scale (VAS) and an improvement in ROM of at least 20° in forward flexion or external rotation compared to baseline at the 12-week follow-up.

Methods: This quasi-experimental study was conducted at the Orthopedic and Spine Department, Khyber Teaching Hospital, Peshawar, from January 5, 2024, to July 5, 2024. A total of 148 patients of both genders with adhesive capsulitis were included. After using aseptic technique, a solution containing 80 mg/mL Depo-Medrol and 2% lignocaine was injected into the affected shoulder joint via a posterior approach using a blind technique. Patients were followed weekly for 12 weeks. Efficacy was assessed at the end of the follow-up period, based on pain relief and improvement in range of motion. No adjuvant therapy, such as physiotherapy, was administered during the study period.

Results: The mean age of the patients was 48.99±10.03 years, with a mean BMI of 25.07±2.06 kg/m², a baseline VAS score of 6.12±1.21, and an average symptom duration of 13.17±5.06 weeks. At the end of the 12-week follow-up period, efficacy was defined as pain relief and improvement in range of motion, which were achieved in 53.4% of patients.

Conclusion: Intra-articular corticosteroid injections are moderately effective in the treatment of adhesive capsulitis, with just over half of the patients experiencing positive outcomes.

## Introduction

Adhesive capsulitis (AC), also commonly known as frozen shoulder, is a chronic, painful condition affecting the shoulder. There is progressive fibrosis and contracture of the capsule of the glenohumeral joint, which results in pain and stiffness [1]. It has a prevalence of 2-5% in the common population, but a prevalence of 11-30% in diabetic patients [2,3]. The middle-aged population is most commonly affected, and it occurs mostly in women between 50 and 60 years of age [4].

Adhesive capsulitis is divided into the following three clinical phases: the painful, adhesive (frozen), and recovery (thawing) phases [5]. Adhesive capsulitis is usually diagnosed clinically. There is pain and limitation in both active and passive ranges of movement, with the most restriction in external rotation [6]. The treatment of adhesive capsulitis is controversial, with several treatment options, none of which is superior to the others [7]. These include physiotherapy, nonsteroidal anti-inflammatory drugs (NSAIDs), oral corticosteroids, intra-articular corticosteroid injections, hydraulic joint capsule distension, manipulation under anesthesia, and arthroscopic release [8,9].

Intra-capsular corticosteroid injection is an effective conservative treatment modality in adhesive capsulitis. When given in the early stages of adhesive capsulitis, it rapidly reduces pain, prevents fibrosis, and shortens the duration of the disease [10]. Blanchard et al. found no significant improvement with physical activity and were of the opinion that intra-articular corticosteroid injection is more effective compared to other forms of treatment [11].

A study by Song et al. found that efficacy was 44.1% following intra-articular injection of corticosteroid in patients with adhesive capsulitis [12]. Most studies on the efficacy of corticosteroid injections have been conducted in Western populations, with limited data from South Asian cohorts. Given the limited availability of regional data and the possibility that variations in health systems, access to care, and patient comorbidities may influence outcomes, it cannot be assumed that findings from international studies directly apply to our local population. The lack of locally generated evidence creates a knowledge gap, particularly regarding the optimal timing, dosage, and delivery method for corticosteroid injections to achieve maximal pain relief and functional improvement. By generating data from our patient population, this study aimed to provide guidance for the first-line therapy recommendations for adhesive capsulitis in this setting, providing orthopedic surgeons with region-specific evidence and supporting more standardized and appropriate patient care.

## Materials and methods

This quasi-experimental study was conducted at the Orthopedic and Spine Department, Khyber Teaching Hospital, Peshawar, from January 5, 2024, to July 5, 2024. The purpose, procedure, risks, and benefits of the study were explained in detail to the patients. Confidentiality was assured prior to obtaining informed consent. After consenting, the eligible patients were enrolled in the study. Information about patients, including age, gender, BMI, baseline visual analog scale (VAS) score, side of shoulder, family socioeconomic status, education, residential status, and duration of symptoms, was recorded.

Adhesive capsulitis was diagnosed according to the International Society of Arthroscopy, Knee Surgery, and Orthopaedic Sports Medicine (ISAKOS) criteria, which include shoulder pain persisting for at least one month, restricted passive motion in at least two planes, including external rotation, and no radiographic evidence of other shoulder disorders. In our study, this corresponded to an insidious onset of shoulder pain with a visual analog scale (VAS) score >4, accompanied by a global limitation of both active and passive range of motion, defined as forward flexion <100°, external rotation 0°-20°, and internal rotation below the thoracic vertebral level. While we acknowledge that advanced imaging, such as MRI, can enhance diagnostic accuracy and help exclude secondary causes of shoulder stiffness, MRI was not performed due to limited resources. Diagnosis was based on clinical examination in accordance with ISAKOS criteria, supported by plain radiographs to exclude structural pathologies. Using aseptic technique, a solution containing 80 mg/mL Depo-Medrol and 2% lignocaine was injected into the affected shoulder joint via a posterior approach using a blind technique. Patients were followed weekly for 12 weeks, and efficacy was defined as pain relief exceeding the minimal clinically important difference (MCID) on VAS, and improvement in range of motion in at least two planes was assessed at the final follow-up. No adjuvant therapy, such as physiotherapy, was administered during the study period.

The estimated sample size was 148, calculated using the WHO sample size formula. This formula takes into account the anticipated proportion of 44.1% for adhesive capsulitis, a 95% confidence level, and an 8% margin of error, utilizing a non-probability consecutive sampling technique [13]. The inclusion and exclusion criteria are presented in Table 1.

**Table 1:** Inclusion and exclusion criteria. Note: The diagnosis was made clinically according to the criteria of the International Society of Arthroscopy, Knee Surgery, and Orthopaedic Sports Medicine (ISAKOS), which require shoulder pain persisting for at least one month, restricted passive motion in at least two planes (including external rotation), and the exclusion of other shoulder disorders on radiographs.

Inclusion criteria	Exclusion criteria
Patients aged 30-70 years	History of shoulder trauma or surgery
Either gender	Documented osteoporosis
Diagnosed with adhesive capsulitis (see note below for operational definition)	Degenerative shoulder conditions (e.g., rotator cuff tear)
Visual analogue scale (VAS) score >4 at presentation	Prior intra-articular corticosteroid injections
-	Concurrent diagnoses, such as reflex sympathetic dystrophy, neuromuscular disorders, cervical radiculopathy, or referred shoulder pain

Documented osteoporosis is excluded because it can independently cause pain, reduced shoulder function, and altered biomechanics, potentially confounding assessment of pain relief and range of motion following corticosteroid injection. It also carries a higher risk of microfractures or complications during shoulder mobilization. Patients over 70 years were also excluded to minimize confounding from age-related degenerative changes in the shoulder joint (e.g., osteoarthritis, rotator cuff degeneration) and systemic comorbidities, which could independently affect pain, range of motion, and recovery following corticosteroid injection. Additionally, older patients are more likely to have multiple coexisting conditions, reduced healing potential, and altered pain perception, making it difficult to attribute observed outcomes solely to the intervention.

Data analysis

The data analysis was performed using SPSS version 24 (Armonk, NY: IBM Corp.). Mean±standard deviation (SD) or median was used for quantitative variables, such as age, BMI, baseline VAS score, and duration of symptoms. Frequencies and percentages were calculated for qualitative variables, including gender, side of shoulder, family socioeconomic status, education, residential status, and efficacy. Efficacy was defined as a reduction in pain score and improvement in range of motion at the end of the 12-week follow-up period compared to baseline. Pain was assessed using the visual analog scale (VAS), and range of motion was assessed clinically using a goniometer. Effect modifiers, such as age, gender, BMI, side of shoulder, baseline VAS score, socioeconomic status, education, residential status, and duration of symptoms, were controlled through stratification. Post-stratification, the chi-square test was applied, and a p-value ≤0.05 was considered statistically significant.

## Results

The study included 148 patients aged between 30 and 70 years, with a mean age of 48.99±10.03 years and an average BMI of 25.07±2.06 kg/m². The baseline visual analog scale (VAS) score was 6.12±1.21, and the mean duration of symptoms was 13.17±5.06 weeks. The majority of participants were female (90, 60.8%) and had right-sided shoulder involvement (83, 56.1%). Regarding socioeconomic status, 66 (44.6%) patients belonged to the middle-income group, while 51 (34.5%) and 31 (20.9%) were from low- and high-income groups, respectively. Educationally, most had primary education (63, 42.6%), followed by secondary (39, 26.4%), uneducated (29, 19.6%), and higher education (17, 11.5%). A slightly more patients resided in urban areas (82, 55.4%) compared to rural settings (66, 44.6%). Treatment was reported as effective in 79 (53.4%) cases, while 69 (46.6%) patients did not experience a significant benefit (Table 2).

**Table 2:** Demographic and clinical profile of patients. Continuous variables are presented as mean±standard deviation (SD). Categorical variables are presented as frequency (n) and percentage. No statistical tests were applied in this table. Statistical significance was defined as p≤0.05, where applicable, in subsequent analyses. VAS: visual analog scale

Categories	Variables	Mean±SD	Frequency (n)	Percentage
Demographics	Age (years)	48.99±10.03	-	-
	BMI (kg/m²)	25.07±2.06	-	-
	Baseline VAS score	6.12±1.21	-	-
	Duration of symptoms (weeks)	13.17±5.06	-	-
Gender	Male	-	58	39.2%
	Female	-	90	60.8%
Side of shoulder	Left	-	65	43.9%
	Right	-	83	56.1%
Socioeconomic status	Low	-	51	34.5%
	Middle	-	66	44.6%
	High	-	31	20.9%
Education level	Uneducated	-	29	19.6%
	Primary	-	63	42.6%
	Secondary	-	39	26.4%
	High	-	17	11.5%
Residential status	Rural	-	66	44.6%
	Urban	-	82	55.4%
Efficacy	Yes	-	79	53.4%
	No	-	69	46.6%

Subgroup analysis of treatment efficacy defined as a reduction in pain score on the visual analog scale (VAS) exceeding the minimal clinically important difference (MCID) of 1.5 points, together with improvement in range of motion at the 12-week follow-up revealed no statistically significant associations between demographic or clinical variables and treatment outcome (p>0.05 for all). Among the 83 patients aged 30-50 years, 47 (56.6%) achieved efficacy compared to 32 of 65 patients (49.2%) aged 51-70 years. Male patients demonstrated greater improvement, with 34 of 58 (58.6%) responding compared to 45 of 90 (50.0%) females (p=0.305). Efficacy was nearly identical between patients with BMI <25 kg/m², 40 of 75 (53.3%), and those with BMI ≥25 kg/m², 39 of 73 (53.4%). Side of shoulder involvement showed no significant effect, with 37 of 65 (56.9%) left shoulder cases and 42 of 83 (50.6%) right shoulder cases responding.

Baseline pain severity also did not significantly influence outcomes among patients with VAS score of 4-6; 36 of 70 (51.4%) achieved efficacy, compared to 43 of 78 (55.1%) with a VAS of >6 (p=0.652). By socioeconomic status, efficacy was observed in 27 of 51 (52.9%) low-income, 32 of 66 (48.5%) middle-income, and 20 of 31 (64.5%) high-income patients (p=0.355). Education level showed a non-significant trend of increasing efficacy with higher education, 15 of 29 (51.7%) in the uneducated group, 32 of 63 (50.8%) in the primary group, 21 of 39 (53.8%) in the secondary group, and 11 of 17 (64.7%) in the higher education group (p=0.782). Duration of symptoms showed a modest, non-significant difference in efficacy, with 36 of 73 (49.3%) for symptoms <12 weeks versus 43 of 75 (57.3%) for >12 weeks (p=0.328) (Table 3).

**Table 3:** Stratification of efficacy of corticosteroids by patient characteristics. Note: "Efficacy yes" refers to patients who achieved a reduction in pain score exceeding the minimal clinically important difference (MCID) of 1.5 points on the VAS and an improvement in range of motion of at least 20° in forward flexion or external rotation at the 12-week follow-up. "Efficacy no" refers to patients who did not meet these criteria. A chi-square test was applied to assess associations between efficacy and categorical variables. A p-value of ≤0.05 was considered statistically significant. VAS: visual analog scale

Variables	Categories	Efficacy yes, n (%)	Efficacy no, n (%)	p-Value
Age (years)	30-50 years	47 (56.6%)	36 (43.4%)	0.371
	51-70 years	32 (49.2%)	33 (50.8%)	
Gender	Male	34 (58.6%)	24 (41.4%)	0.305
	Female	45 (50.0%)	45 (50.0%)	
BMI	<25	40 (53.3%)	35 (46.7%)	0.991
	>25	39 (53.4%)	34 (46.6%)	
Side of the shoulder	Left	37 (56.9%)	28 (43.1%)	0.444
	Right	42 (50.6%)	41 (49.4%)	
VAS score	4-6	36 (51.4%)	34 (48.6%)	0.652
	>6	43 (55.1%)	35 (44.9%)	
Socioeconomic status	Low	27 (52.9%)	24 (47.1%)	0.355
	Middle	32 (48.5%)	34 (51.5%)	
	High	20 (64.5%)	11 (35.5%)	
Education level	Uneducated	15 (51.7%)	14 (48.3%)	0.782
	Primary	32 (50.8%)	31 (49.2%)	
	Secondary	21 (53.8%)	18 (46.2%)	
	High	11 (64.7%)	6 (35.3%)	
Residential status	Rural	35 (53.0%)	31 (47.0%)	0.939
	Urban	44 (53.7%)	38 (46.3%)	
Duration of symptoms	<12 weeks	36 (49.3%)	37 (50.7%)	0.328
	>12 weeks	43 (57.3%)	32 (42.7%)	

## Discussion

The mean age of the patients was 48.99 years, which is consistent with the typical age range for adhesive capsulitis, a condition most commonly seen in middle-aged individuals and thought to result from capsular inflammation and fibrosis leading to progressive stiffness and pain. The mean BMI of 25.07 kg/m² reflects a normal to slightly elevated body weight. BMI was recorded as a potential effect-modifying factor because excess body weight can influence shoulder biomechanics and is often associated with metabolic conditions, such as diabetes, that have been linked to adhesive capsulitis, but it isn't extreme enough to suggest obesity as a major contributing factor in this cohort. The baseline VAS score of 6.12 indicates moderate to severe pain, which aligns with the characteristic pain intensity associated with adhesive capsulitis, typically driving patients to seek treatment. The symptom duration of 13.17 weeks suggests that patients presented with a subacute condition, likely reflecting its chronic nature before treatment was sought.

The gender distribution, with 90 (60.8%) females and 58 (39.2%) males, reflects the known higher prevalence of adhesive capsulitis in females, possibly due to hormonal differences, particularly post-menopausal changes that affect connective tissue elasticity and joint health [14]. In terms of the affected shoulder, the slightly higher incidence of right shoulder involvement (56.1%) could be explained by the dominance of the right arm in the general population, leading to greater use and mechanical stress on the joint, which may contribute to the development of adhesive capsulitis through inflammation and subsequent capsular fibrosis.

Socioeconomic status showed that the majority of patients came from low to middle socioeconomic backgrounds, which could be related to greater physical labor in these groups, as well as potential delays in accessing healthcare, leading to more advanced or chronic presentations by the time treatment is sought. Education level analysis showed a non-significant trend of increasing efficacy with higher educational attainment, with efficacy rates of 51.7% for uneducated patients, 50.8% for those with primary education, 53.8% for those with secondary education, and 64.7% for those with higher education. These differences were not statistically significant (p=0.782), and no causal explanation is inferred, as this association was not evaluated in the study design.

In terms of residential status, a slightly higher percentage of patients came from urban areas. This could be due to better access to healthcare services in urban regions, resulting in more diagnoses and treatments in these populations. The overall efficacy of treatment, defined as a reduction in pain score and improvement in range of motion at the end of the 12-week follow-up, was 53.4%. All patients received a single intra-articular corticosteroid injection prepared with 80 mg/mL Depomedrol and 2% lignocaine, administered into the affected shoulder joint via a posterior approach under aseptic technique. The injections were performed by an orthopedic surgeon experienced in shoulder procedures. No additional treatments, such as physiotherapy, were provided during the study period to ensure that the outcomes could be attributed solely to the injection. The stratified analysis by age showed no significant difference in efficacy between younger (30-50 years) and older (51-70 years) patients, suggesting that the response to treatment was not strongly age-dependent in this cohort. The observed treatment efficacy was 58.6% in males and 50.0% in females. This difference did not reach statistical significance (p=0.305), and no causal inference is made, as potential underlying factors were not assessed in this study and were not part of the baseline variables.

BMI did not show a significant effect on efficacy, indicating that moderate variations in body weight did not significantly influence the treatment response. This suggests that factors other than BMI may play a larger role in determining outcomes for adhesive capsulitis. The similar efficacy rates between left and right shoulders suggest that side dominance does not significantly affect the response to treatment, even though right shoulders are more frequently affected.

The stratification by baseline VAS score indicated no significant difference in efficacy, suggesting that the level of pain at the onset does not predict the success of treatment. This implies that the biological mechanisms driving pain and stiffness might be more critical to recovery than the intensity of pain alone. The socioeconomic stratification revealed that patients from higher socioeconomic backgrounds had a higher observed rate of treatment efficacy (64.5%) compared to those from middle (48.5%) and lower (52.9%) socioeconomic groups. This finding represents a trend within our data; however, it did not reach statistical significance (p=0.355), and no causal explanation was confirmed within the scope of this study. As this association was not part of our baseline hypothesis and has not been extensively evaluated in our local population, we have refrained from attributing it to specific factors. Education level did not significantly impact efficacy; however, patients with higher education tended to have slightly better outcomes, potentially due to a better understanding and adherence to post-treatment care and rehabilitation.

Lastly, patients with symptoms lasting more than 12 weeks had a slightly better response (57.3%) compared to those with shorter symptom duration. This could indicate that patients with longer-standing symptoms might experience more notable improvement, as the condition could have progressed to a stage where the intervention offers more dramatic relief. However, this difference did not reach statistical significance (p=0.328), and no causal explanation is inferred, as this association was not evaluated in the study design and was not part of the baseline hypothesis.

Lee et al. conducted a randomized trial comparing corticosteroid injections in the coracohumeral ligament (CHL) and inferior glenohumeral capsule (IGHC) with injections into the posterior glenohumeral recess (PGHR) for adhesive capsulitis (AC). A total of 120 patients with AC were enrolled and followed over four months. The study demonstrated significant improvements in pain (as measured by VAS reduction from baseline) and range of motion (ROM) in both groups at two and four months. However, the CHL+IGHC group showed significantly better outcomes in pain relief and ROM compared to the PGHR group. The CHL+IGHC group required fewer injections (2.58±0.59) than the PGHR group (2.93±0.25). The study concluded that the CHL+IGHC injection approach is superior to the PGHR approach in terms of pain relief and functional improvement in adhesive capsulitis [15].

Kim et al. conducted a double-blind randomized controlled trial comparing the efficacy of high-dose (40 mg) versus low-dose (20 mg) triamcinolone acetonide injections in patients with adhesive capsulitis. Thirty-two patients with severe pain (Numeric Rating Scale {NRS} >8) were randomly assigned to either the high-dose or low-dose group. The primary outcome measures were pain (NRS) and function (Shoulder Pain and Disability Index {SPADI}). Both groups exhibited significant improvements in NRS (p=0.01) and SPADI scores (p=0.02 for low-dose; p<0.01 for high-dose), with no significant differences between the groups. The study concluded that 20 mg of triamcinolone is adequate for significant pain relief and functional improvement, making it a sufficient dose for treating adhesive capsulitis [16].

Wang et al. conducted a meta-analysis that included five randomized controlled trials (RCTs) evaluating corticosteroid injections for adhesive capsulitis. The analysis comprised 225 patients in total, and the results demonstrated that intra-articular corticosteroid injections significantly improved pain and ROM at 0 to eight weeks. However, the effects on pain relief did not persist beyond 24 weeks, though ROM improvements were still observed. The study suggested that while corticosteroid injections are effective in the short term for pain relief, long-term benefits may be limited primarily to improvements in ROM [17].

Sharma et al. conducted a randomized controlled trial comparing corticosteroid injections with and without distension in patients with adhesive capsulitis. The study involved 106 patients, who were allocated into following three groups: corticosteroid injections alone, corticosteroid with distension, and a control group receiving standard care. Significant improvements were observed in the intervention groups in terms of pain relief (Numeric Pain Rating Scale {NPRS}) and function (SPADI) at eight weeks compared to the control group. However, there were no significant differences between the two intervention groups. At 12 months, all groups, including the control group, showed similar outcomes, reflecting the natural history of adhesive capsulitis [18].

Griesser et al. conducted a systematic review evaluating the effectiveness of intra-articular corticosteroid injections for adhesive capsulitis, synthesizing data from 10 randomized controlled trials involving a total of 852 patients. The review demonstrated that corticosteroid injections were significantly more effective than placebo in improving pain and range of motion (ROM) in the short term. However, at 12-24 weeks post-injection, outcomes, such as pain relief and functional recovery, were comparable between corticosteroid and physiotherapy interventions [19].

Limitations

This study has several limitations. First, the follow-up period was limited to 12 weeks, which may not adequately capture the long-term effects, recurrence rates, or the complete natural history of adhesive capsulitis. Second, a blind posterior approach was used for intra-articular corticosteroid injection. Third, the absence of a control or comparison group restricts the ability to evaluate the relative efficacy of corticosteroid injection against other conservative interventions. Fourth, the study was conducted in a single tertiary care centre, which may limit the generalizability of the findings to other populations and healthcare systems. Finally, data on comorbidities, including diabetes and hypothyroidism, were not collected in the original study design and therefore could not be incorporated into baseline characteristics. These comorbidities may influence the prognosis of adhesive capsulitis.

Recommendations

Future research should include longer follow-up durations to assess sustained outcomes, compare image-guided versus blind injection techniques, and evaluate corticosteroid injection against other conservative and surgical interventions. Multi-centre, randomized controlled trials in diverse populations are recommended to strengthen the evidence base and guide standardized treatment protocols for adhesive capsulitis.

## Conclusions

Intra-articular corticosteroid injection demonstrated a positive treatment response defined as pain reduction and improved range of motion in just over half of patients with adhesive capsulitis. The treatment effect was consistent across demographic and clinical subgroups, with no statistically significant influence from age, gender, BMI, side affected, socioeconomic status, education level, or symptom duration. These findings support the use of corticosteroid injection as a useful conservative intervention for adhesive capsulitis in our patient population.
